# Subject-Specific Effect of Metallic Body Accessories on Path Loss of Dynamic on-Body Propagation Channels

**DOI:** 10.1038/srep29818

**Published:** 2016-07-20

**Authors:** H. A. Rahim, M. Abdulmalek, P. J. Soh, K. A. Rani, N. Hisham, G. A. E. Vandenbosch

**Affiliations:** 1 Bioelectromagnetics Research Group (BioEM), School of Computer and Communication Engineering, Universiti Malaysia Perlis (UniMAP), Pauh Putra, Arau, Perlis 02600, Malaysia; 2 Department of Engineering and Information Science, University of Wollongong in Dubai, Dubai, UAE; 3 Advanced Communication Engineering (ACE) CoE, School of Computer and Communication Engineering, Universiti Malaysia Perlis (UniMAP), Pauh Putra, Arau, Perlis, 02600 Malaysia; 4 ESAT-TELEMIC, Katholieke Universiteit Leuven, Kasteelpark Arenberg 10, Leuven 3001, Belgium; 5 School of Electrical System Engineering, Universiti Malaysia Perlis (UniMAP), Pauh Putra, Arau, Perlis 02600, Malaysia

## Abstract

This paper presents the investigation of path loss variation for subject-specific on-body radio propagation channels, considering the effect of metallic spectacles and loop like metallic accessories. Adding metallic items may affect the operability of Body Centric Wireless Communications (BCWC). Measurements were carried out in an RF-shielded room lined with microwave absorbing sheets for strategically placed bodyworn antennas covering the upper front torso and the lower limbs. The path loss of the on-body radio channel was characterized explicitly taking into account the body size of the subjects. For metallic loop-like accessories, the results indicate that for underweight subjects, there was a slightly higher influence, up to 2%, compared to normal and overweight subjects. Our findings indicate that a noticeable effect exists on on-body channels for dynamic movements where the metallic watch acts as a local scatterer that affects the non-line-of-sight (NLOS) signal path between transmitter and receiver for underweight subjects in comparison to normal and overweight subjects. The path loss decreases when the receiving terminal was positioned very close to the metallic item. If a loop-like metallic accessory is not appropriately considered when designing the radio channel on a subject, the reliability of the body-centric wireless system may degrade.

The revolution of Body Centric Wireless Communications (BCWC) in the past few years perfectly fits within the paradigm of “anytime-anywhere personalized telecommunications.” Specific applications are related to specific occupations, e.g., paramedic personnel and fire fighters, military personnel, and in the field of multimedia, sports and fitness^1^,^2^,^3^,^4^,^5^,^6^,^7^. The miniaturization of wearable hardware, embedded software, digital signal processing etc. has turned human to human networking integrated with wearable sensors into reality^1^,^2^. In wireless medical communications^7^,^8^,^9^, for example, wearable health monitoring systems are implemented using radio technology as BCWC offers greater mobility and provides comfort to patients by freeing them from physical connections to hospital equipment. This thanks to the fact that BCWC is capable of continuously and remotely monitoring a patient’s vital physiological data, including heart rate, blood pressure, respiratory rate, and body temperature, without interrupting their normal activities^4^. Normally, BCWC includes a number of wireless sensors placed on the human body or implanted that communicate with each other, other on-body units, or external base stations^6^.

A key characteristic in these systems is the path loss, which represents the attenuation of the signal. It is defined as the ratio between the transmitted and received power^10^. The performance of any wireless link can be assessed based on the prediction of the average received signal strength as a function of distance. This can be achieved through a path loss model that enables the system designers to predict the signal-to-noise (SNR) for a mobile communication system^11^, such as body-centric wireless communications. Such path loss models are known as large-scale propagation models. The output of these models is the estimation of the path loss as a function of distance. It is investigated in this work as the main criterion. The performance of BCWC is strongly dependent on the environment in which the communication between the transmitting and receiving antennas takes place. Due to the human body characteristics that differ in terms of dimensions and tissue properties, the BCWC is greatly affected when such system operates in very close proximity to the human body. This will directly influence the path loss of the system, consequently producing an erroneous computation of the system link budget^12^. In addition to the human body’s characteristics, other factors also contribute to altering the characteristics of the on-body radio channel. These other factors include the posture of the body, dynamic movement of the body^13^, frequency of operation, the antenna polarization, and presence of metallic objects on the body. If these factors are not considered appropriately when designing the radio channel, significant degradation of the system performance could occur.

A significant amount of research has been conducted and reported in the open literature on on-body radio propagation^1^,^2^,^3^,^4^,^5^,^10^,^12^,^13^,^14^,^15^,^16^,^17^,^18^,^19^,^20^,^21^,^22^,^23^,^24^,^25^,^26^,^27^,^28^,^29^,^30^,^31^,^32^,^33^,^34^,^35^,^36^,^37^,^38^,^39^. Some of the research dealt with body-to-body propagation^14^,^15^,^16^,^17^,^18^, and other research dealt with on-body to in-body propagation^19^. The effect of movement on the narrowband on-body communication channel has been investigated in^2^,^13^,^17^,^20^,^21^,^22^,^23^,^24^. In^21^, the authors reported that a Nakagami-m distribution well described on-body mobile channels. The characterization of an on-body propagation channel at 2.45 GHz based on path loss in an indoor environment was presented in^24^, proposing a novel approach to model a channel with an autoregressive transfer function. A path loss model for an on-body channel at 2.45 GHz in an indoor environment based on simulation and measurement results, as well as a model of the power delay profile was discussed in^2^. A numerical, transient, and statistical analysis of an ultra-wideband (UWB) on-body channel, considering different body positions under static and/or dynamic conditions, was performed in^4^,^5^,^10^,^12^,^25^,^26^,^27^,^28^,^29^,^30^,^31^. In^32^,^33^, a spatial diversity technique was utilized and demonstrated in order to enhance the performance of BCWC. Very few publications address the effect of subject specific features on the path loss characteristics^12^,^34^,^35^,^36^,^37^. A recent publication^37^ proposed two linear models to estimate the effect of subject specificity for LOS and NLOS scenarios based on parallel finite-difference time-domain (PFDTD) numerical calculations at 5.8 GHz. However, in the NLOS case, only one on-body link was considered, i.e., at the waist. As it is inevitable that metallic accessories such as spectacles and watches are worn on the human body, their placement directly or indirectly in the propagation path of the transmission will impact the on-body radio channel characteristics. In addition, due to the nature of the human body, which involves various movements, this radio channel is more difficult to characterize in the presence of metallic accessories. Despite its importance, evaluation of the effect of metallic items worn on the body has received very little attention in the literature. Due to the body size variations, consideration of the specific subject and her or his movements are required to provide a better understanding of the characteristics of narrowband on-body communications.

In the past, several studies were conducted to investigate the effects of metal objects in close proximity to the radio frequency radiating source, both for radio frequency identification (RFID) systems^38^ and mobile phones, on antenna impedance matching as well as specific absorption rate (SAR) distribution^39^,^40^,^41^, respectively. The effects of metallic objects on antenna matching and gain, as well as SAR distribution, were clearly demonstrated. However, to the best of our knowledge, a thorough subject-specific experimental investigation of the influence of on-body worn metallic items on the on-body propagation channel has not been reported yet in open literature. The results reported further in this paper also clearly demonstrate specificity depending on the physical and other characteristics of the subject.

## Measurement Setup

**Figure 1:**
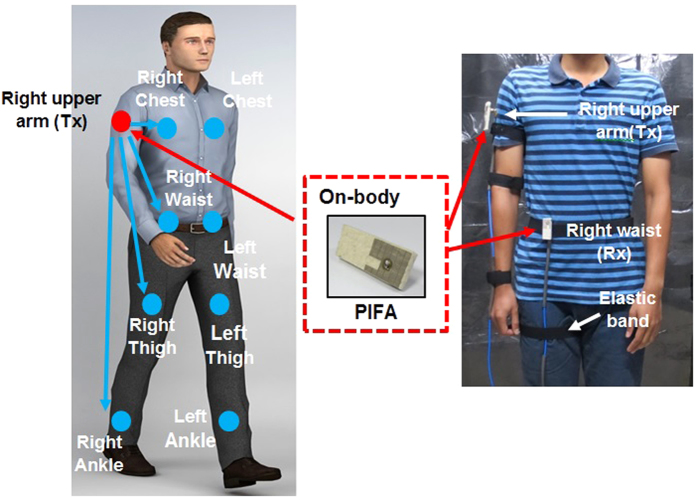
Measurement setup for narrowband channel on-body characterization (**a**) Tx and Rx locations on-body (**b**) measurement in an RF shielded room^45^.

An extensive on-body measurement campaign was performed in an RF-shielded facility lined using microwave absorbing sheets in the High Voltage laboratory, School of Electrical System Engineering, Universiti Malaysia Perlis, Perlis, Malaysia. The chamber had a floor area of 8.9 m^2^. This measurement campaign aimed at investigating the effect of metal on the radio propagation channel for on-body scenarios at 2.45 GHz, see Fig. 1 A 2-port Vector Network Analyzer (PNA) (Agilent PNA E8362B) was utilized to capture the *S*_*21*_ of a link with transmitting (Tx) and receiving (Rx) antennas placed on the body. The PNA was configured with an output power of 0 dBm and it was consistently calibrated. At a single carrier frequency *f*_*c*_ = 2.45 GHz, using a sampling time interval of 10 ms and a total number of sampled points per acquisition N = 1601, a total of 32020 points for each link was collected. Two four-meter low loss semi-flexible Huber Suhner coaxial cables were used. These cables were wrapped with microwave absorbing foam (Eccosorb Flexible Broadband Urethane Absorber number FGM-U-20-SA) to minimize the spurious radiation from the coaxial cable and the coupling between them. This measurement setup followed the standard procedure reported in^13^,^32^,^33^.

Six subjects were considered, three male and three female, with body weights that ranged from 35 to 110 kg. The most important features of the test subjects, i.e., height, weight, body mass index (BMI), and the circumferences of a few significant body parts are presented in Table 1. The study was approved by the Ethics Committee of the university, Universiti Malaysia Perlis (Perlis, Malaysia). The measurements were conducted in accordance with the approved guidelines. Written informed consent was obtained from all participants. The subjects were categorized into three distinct groups, based on their body size, according to the World Health Organization’s (WHO’s) BMI classification for adults: underweight (UW) (BMI < 18.50), normal (18.50 ≤ BMI ≤ 24.99), and overweight (OW) (BMI ≥ 25.0).

**Figure 2:**
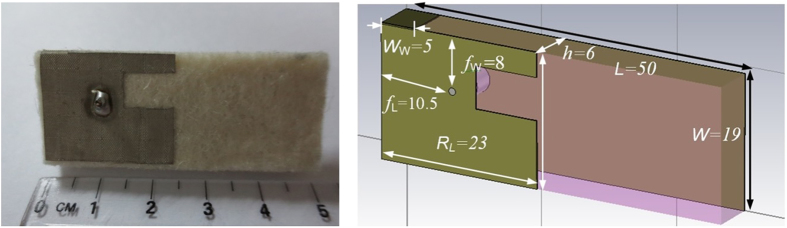
Structure and dimensions of the utilized PIFA (front view)^43^

In this work, the antennas are considered to be part of the on-body radio propagation channel. Although monopole antennas placed normally to the body with a relatively high body-antenna separation will result in a reduced path loss^42^, such on-body mounting configurations are less practical for WBAN applications compared to small, low-profile antennas worn directly on the body. Therefore, a pair of low-profile wearable linearly polarized slotted planar inverted-F antennas (PIFA) operating in the 2.45 GHz Industrial Scientific Medical (ISM) band was utilized^43^, see Fig. 2. PIFAs are good candidates for WBAN applications^13^, and can easily be placed conformal to the body to with a reasonably good link performance. Also, PIFAs are less affected by the human body proximity compared to other types of omnidirectional antennas, as the presence of the full ground plane in PIFAs shields the radiating element from the lossy human tissues^44^. It was observed that the changes on the antenna’s matching and radiation patterns of PIFA are almost negligible due to the presence of the full ground plane.

The ground plane and the radiating patch of each antenna are made out of Shieldit Super from Less EMF Inc and a thick felt (6 mm) is used as substrate (*ε*_*r*_ = 1.43). The PIFAs were placed directly on the subject’s body with a distance *d* of 10.1 mm between the subject’s clothing (which is 1 mm thick) and the PIFA’s ground plane. More details on the performance and characteristics of these specific antennas can be found in^43^. Both the transmitter and the receiver antennas were both vertically-oriented (with the radiator placed on the top section) when mounted on the subject’s body. The Tx (connected to port 1) was fixed at the right side of the upper arm (RUA), close to the shoulder. This location is more realistic for BCWC applications, since it does not limit the user’s regular movements. Moreover, this location may represent the worst case scenario when a non-line-of-sight (NLOS) propagation path is considered, compared to the widely-investigated transmitter placement on the chest.

The Rx (connected to port 2) was mounted on eight different locations: right chest (RC), left chest (LC), right waist (RW), left waist (LW), right thigh (RT), left thigh (LT), right ankle (RA), and left ankle (LA). The distances between Tx and all Rx for each subject are listed in Table 2. In order to avoid direct near-field coupling between Tx and Rx, it was ensured that the Tx-Rx separation was greater than *λ*/*2*. Figure 1 illustrates the locations of the Tx and Rx antennas. Although the environment was quite dynamic, best efforts have been taken to ensure consistency, i.e., the Tx and Rx positions were set, and the position of the Tx and Rx cables were routed and fixed around the subjects’ bodies in the same way. The measured data in this static scenario were captured when the signal reached the quasi steady state mode, with minimum signal variation in the *S*_*21*_ values. Placements of the Tx and Rx(s) were performed relative to the center of the tested body part. Throughout our validation, this position can only vary by no more than ± 0.5 cm. The subjects also were asked to remove all metallic items they were wearing on their bodies.

**Figure 3:**
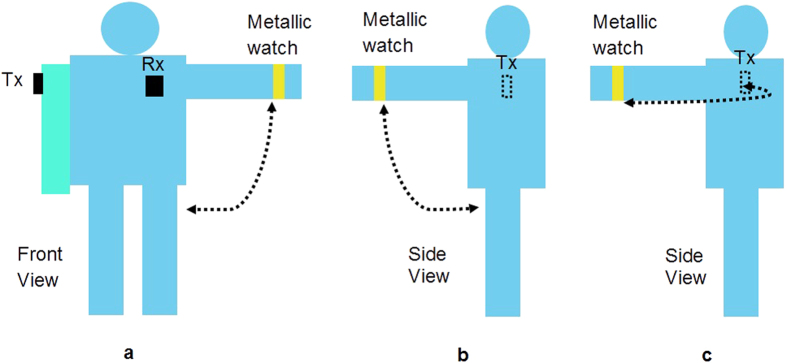
Arm with metallic watch moves (**a**) to the side of body (**b**) side view: to front of body and back to initial position and (**c**) side view: from front to left side and return to initial position.

Two sets of measurements have been performed. In the first experiment, the subjects were in standing position with their arms stretched alongside the body. In the second set of measurements, the subjects were performing random movements for each on-body link. The Tx and Rx(s) were maintained in the same on-body positions as in the first experiment. Two categories of these random movements were considered, representing daily routine movements: arm movements and body movements. The body movements are listed in Table 3. The random arm movements, with the metallic accessory worn on the left wrist^30^, were the following:

- Arm along the body move to the side, then moving to the side to a position of 90° with respect to the body trunk, and then returning to the initial position, see Fig. 3(a).
- Arm along the body, then moving forward to the front to a position of 90° with respect to the body trunk and returning to initial the position, see Fig. 3(b).
- Arm in a straight position in front of the body, then moving to the right (90°), and returning to the initial position, see Fig. 3(c).
- Both arms (Tx was moving) swinging forward and returning to the initial position repetitively.

It is assumed that all random movements occurred at a speed of 1.5 m/s, similar as in previous investigation^32^. The sampling time was chosen in such a way that all the variations caused by any fast movement of the body were captured. This was achieved by setting the sampling frequency higher than twice the maximum Doppler shift, *f*_*m*_, where *f*_*m*_ = ν/λ, with λ the free space wavelength and *ν* the (1.5 m/s) average velocity of the movement. The coherence time, *T*_*c*_, for the on-body channels can be estimated from the maximum Doppler shift, *f*_*m*_^11^,


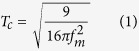


The sampling time is within the coherence time, *T*_*c*_ = 15 ms^32^, ensuring reliable results.

## Results

### Investigated Scenarios

Two distinct metal objects, used in daily life by many people, were investigated:

- Metallic spectacles: full-rimmed and semi-rimmed. The subjects wore the same types of spectacles during the measurements. The spectacles were not in the direct path of propagation and it can thus be expected that their influence is small.
- Three different types of metallic watches: stainless steel, tungsten, and gold-plated 18-carat (K). The subjects were wearing the watch on the left wrist, under two positions: (i) in a standing still position; and (ii) with body and arm movements. This object is much closer to the path of propagation.

### Subject-specific On-body Path Loss Characterization

The path loss exponent, *γ*, is an important propagation parameter in understanding how fast the received power decays with the distance. The path loss is expressed as


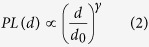


where *d*_*0*_ is the reference distance set at 0.1 m for an on-body channel. The path loss is chosen as the main criterion in this investigation. In the Friis formula, γ = 2 for free space propagation. On-body propagation in a non-reflecting environment depends heavily on various factors including losses of human tissues, creeping waves, and human body reflection, thereby resulting in higher values of path loss exponent compared to free space^10^. A detailed explanation on path loss can be found in^11^.

### Effect of Metallic Spectacles

**Figure 4:**
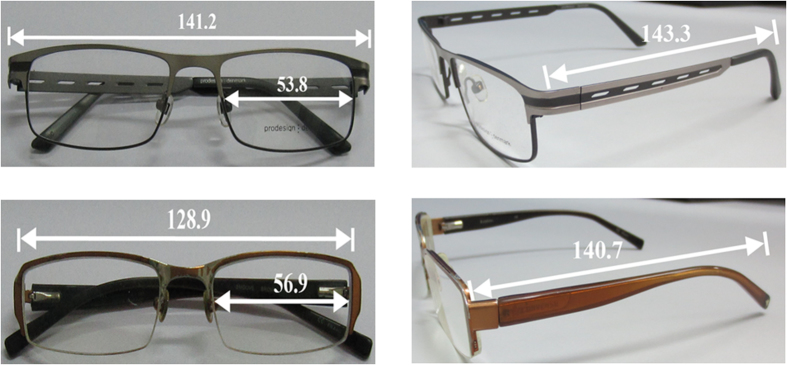
Dimensions of full-rimmed and semi-rimmed spectacles (with all dimensions in millimeters). These photographs were taken by Hasliza A Rahim.

The goal of this investigation is to quantify the effect of spectacles on the on-body propagation channel when they are far from the non-line-of-sight (NLOS) link between Tx and Rx. Due to the location of Tx on the subject’s body, it is obvious that there was no direct line-of-sight (LOS) path such that most of the received power reaches the Rx through a creeping (trapped surface) wave along the human body trunk^21^. It is thus expected that their effect is quite low. Two types of commonly worn metallic spectacles, i.e., full-rimmed and semi-rimmed, were investigated. The spectacles’ dimensions are shown in Fig. 4. The path loss values for the six subjects without any metal accessories and for different locations of the Rx antenna serve as reference. The on-body channel variation caused by respiration was negligible when the subjects were stationary. The dominant component of the creeping wave had greater influence on the variation of the on-body path loss^17^. The mean path loss characterizations are based on the empirical logarithmatic distance path loss model of equation (2). A least square (LS) fit method is applied on the measured data. Table 4 gives the path loss exponent, standard deviation of shadowing and the average path loss. The results obtained in this study for the effect of body size on the path loss exponent without metallic spectacles or watches were close (but not identical) to the results reported in^25^,^26^,^44^: for NLOS, γ ≈ 4.0–4.5, as listed in Table 5. The fact that the path loss exponents in previous work are slightly lower than in this work might be due to the considered types of antennas, polarizations of the antennas, and different considered environment^25^. The measurements also confirm that higher γ values are obtained in NLOS than in LOS^12^,^34^,^36^ for all subjects, which is of course obvious^16^. As expected, it can be seen that the results for γ are quasi the same for the cases with and without spectacles, see Table 4. This confirms that the effect of any reflections at the positions of the metal spectacles is small to very small irrespective of the subject’s body size.

A statistical characterization of the body shadowing, *X*_*σ*_, is also performed for all gathered acquisitions. The measured data were fitted to an empirical distribution, and the cumulative distribution function (CDF) was derived in order to determine the shadowing factor of the on-body radio propagation channel. The body shadowing factor is a zero mean, lognormally distributed statistical variable that considers the deviation of the path losses^20^. The CDF is represented by the *μ* and σ values that refer to the mean and standard deviation of the data, given as


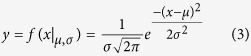


**Figure 5:**
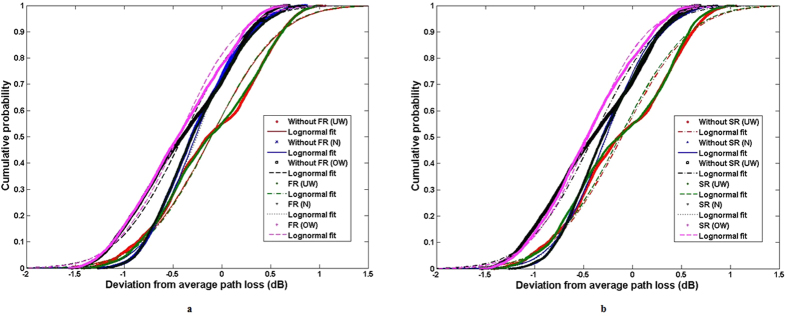
CDF of measured path loss fitted to lognormal distribution without spectacles and wearing (**a**) full-rimmed spectacles (**b**) semi-rimmed spectacles.

Figure 5(a) shows the calculated CDF of the measured path losses for two scenarios: with full-rimmed spectacles and without spectacles. In the case without spectacles, the CDF fits well to the lognormal distribution (Underweight: *μ* = −0.11, *σ* = 0.56, Normal: *μ* = −0.27, *σ* = 0.41, and Overweight: *μ* = −0.40, *σ* = 0.53). Also the CDF when wearing full-rimmed spectacles fits to the lognormal distribution (Underweight: *μ* = 0.10, *σ* = 0.55, Normal: *μ* = −0.25, *σ* = 0.43, and Overweight: *μ* = −0.44, *σ* = 0.50). Figure 5(b) shows the data for the scenario with semi-rimmed spectacles. Finally, also this CDF fits well to the lognormal distribution (Underweight: *μ* = −0.13, *σ* = 0.56, Normal: *μ* = −0.27, *σ* = 0.41 and Overweight: *μ* = −0.45, *σ* = 0.47). These results show that the shadowing factor of the human body was not affected by the presence of both types of spectacles, with approximately the same standard deviation regardless of the subject’s specificity. The very small variation in the dispersion data due to the spectacles was consistent with the path loss exponent associated with the metallic spectacles for all subjects.

### Effect of a Loop-Like Metallic Accessory

**Figure 6:**
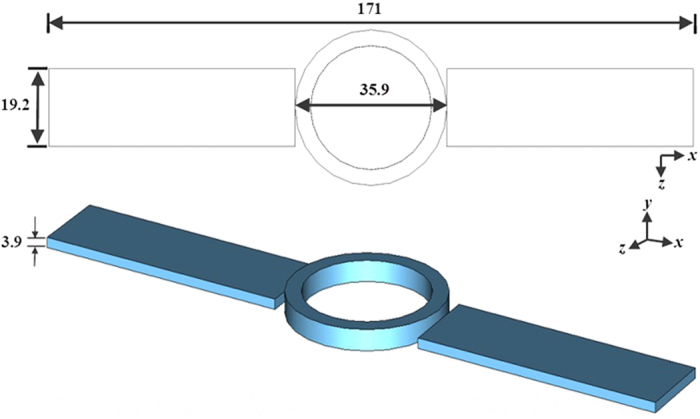
Dimensions of stainless steel watch (all dimensions in millimeters): top view (left) and perspective view (right).

The effect of a loop-like metallic accessory, i.e., a stainless steel, tungsten, or hardmetal gold-plated 18 K watch, was investigated. The key dimensions and conductivities^46^ of the watches are presented in Table 5. Detailed dimensions of the stainless steel watch are shown in Fig. 6. Table 4 shows the path loss exponent values, γ, for different subject groups with and without metallic watches. The results show that the average path loss, *P*(*d*_*0*_), decreases due to the presence of the metallic watch for underweight subjects compared to normal and overweight subjects. For underweight subjects, up to 2% reduction of the average path loss was experienced with a metallic watch. In contrast, the average path loss for normal and overweight subjects remained unchanged in both scenarios, with and without watch. The path loss exponent values were not affected by the presence of the metallic item across the groups. The underweight subjects, who were shorter and thinner compared to other subjects, were more affected by the metallic scatterer reflections, resulting from the shorter communication link. This led to an increase reflection from the metallic watch, in this way affecting signal propagation, suggesting that the *P*(*d*_*0*_) in the presence of the metallic object is subject-dependent. The decrease of *P*(*d*_*0*_) experienced for different types of metallic scatterers by the underweight subjects may also be caused by the different conductivities, as listed in Table 6. Reflections are observed to be higher for higher conductivities. Note that the shapes of the watches used in this experiment were the same, i.e., circular, to ensure the consistency of the results by using consistent dimensional parameters. The thicknesses and widths of the wristbands were different, see Table 6.

**Figure 7:**
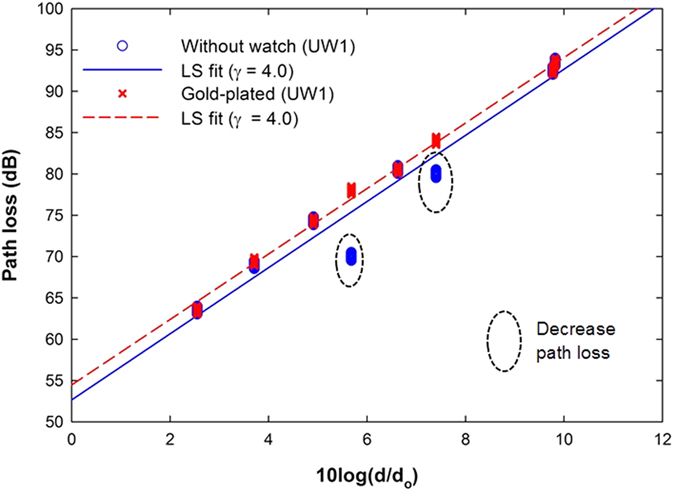
Measured data and path loss model with subject UW1 wearing the gold-plated watch. UW1 shows a decrease of path loss at LW and LT locations.

To further confirm how the metallic scatterer affects the on-body propagation, the result of the individual subject (UW1) is presented in Fig. 7. There are differences in the mean path loss between subject with and without a watch. Relative to the case without watch, the mean path loss for the case with a metallic watch was reduced. Figure 7 shows that the path losses at LW and LT for the UW1 subject were affected by the wave reflected from the metallic watch. The average path loss value, *P*(*d*_*0*_), was reduced by 3.5% and the path losses for RUA-LW and RU-LT links were decreased by an average of about 10.3% and 4.8%, respectively, in comparison to the case without the watch. Figure 7 also shows that there is a higher dispersion of data, with a maximum of about 9 dB at the RUA-LW link for the UW1 subject with a gold-plated 18 K watch, which proves that the on-body propagation was indeed affected by the reflections of the metallic watches. A similar conclusion can be drawn here: the path loss variation is dependent on the specific subject size.

**Figure 8:**
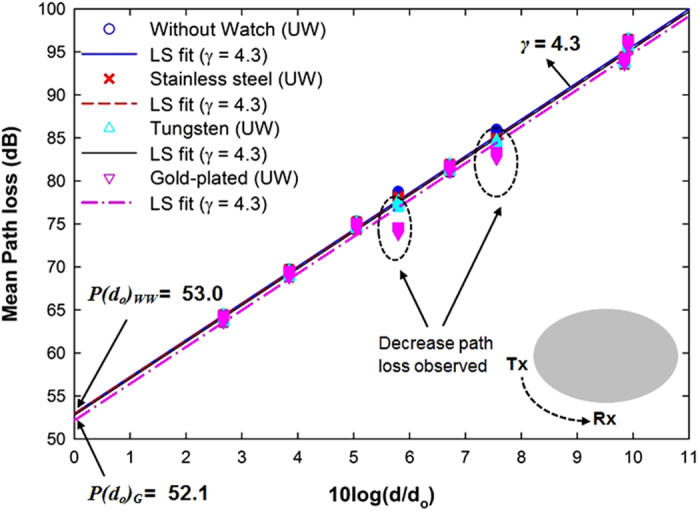
Measured data and path loss model in the scenarios with and without the gold-plated watch for underweight subjects. Reference distances for underweight subjects are the average distance of the on-body links in eight Rx positions (as shown in Table 2).

**Figure 9:**
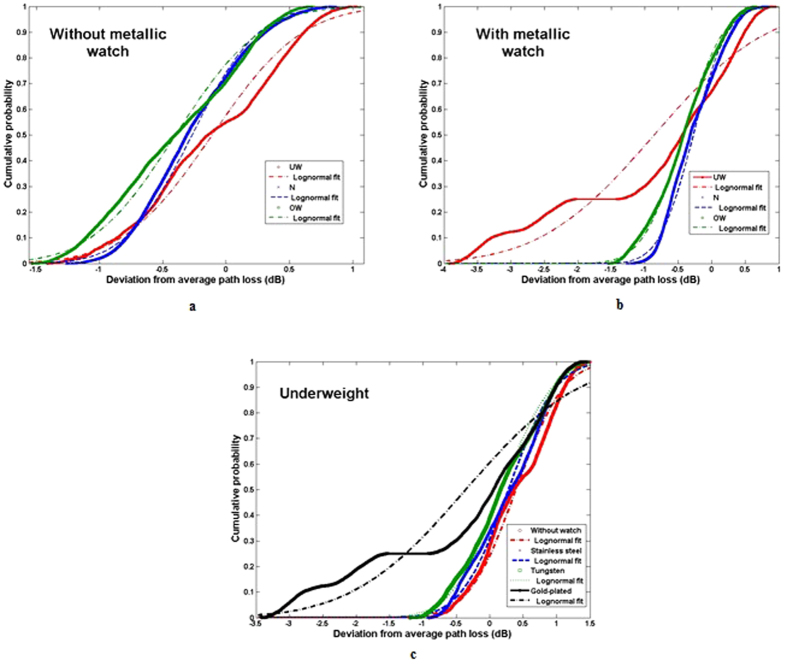
CDF of measured path loss fitted to the lognormal distribution in the scenario with: (**a**) all subjects without metallic watch (**b**) all subjects wearing a gold-plated 18K watch (**c**) Underweight subjects with and without metallic watch. The underweight subjects showed a wider spread of data, indicated by the larger value of *σ, σ* = 1.34.

Figure 8 shows all data acquired for the cases with and without gold-plated 18 K watch (LS fit for underweight subjects). It is quite clear that the effect of the watch is larger than the effect of the spectacles. Generally this is due to the effect of placing metallic accessories along the NLOS path. When the metallic scatterers (watches) are positioned along the NLOS paths, the waves are obstructed, causing the reflections, and this causes larger variations in the path loss values. Figure 9 shows the cumulative distribution of the deviation from the calculated average path loss fitted to the lognormal distribution. The measured data in the case of underweight subjects with stainless steel and tungsten watches (*μ*_*S*_ = −0.22, *σ*_*S*_ = 0.55 and *μ*_*T*_  = −0.32, *σ*_*T*_ = 0.59) fits with the lognormal distribution. The unaffected body shadowing factor of normal and overweight subjects with metallic watches confirms that the path loss variation is subject-specific, i.e., depending on height and weight, as shown in Table 4. Since underweight subjects are shorter and thinner relative to the subjects in the other groups, there is a wider spread of data, indicated by the larger value of *σ, σ*_*G*_ = 1.34, see Fig. 9(c). This most probably is due to the reflected waves from the gold-plated watch, and further supports the conclusion that the path loss of the on-body link for an underweight subject is more susceptible to the presence of a metallic watch worn on his/her body.

### Dynamic Radio Propagation Channels

In a parallel experiment, we further explored the effects of metallic scatterers (watches) for various random body and arm movements to demonstrate the influence of dynamic body motion in the presence of a metallic watch on the performance of the radio channel. The subject was moving at a given pace during the experiment.

**Figure 10:**
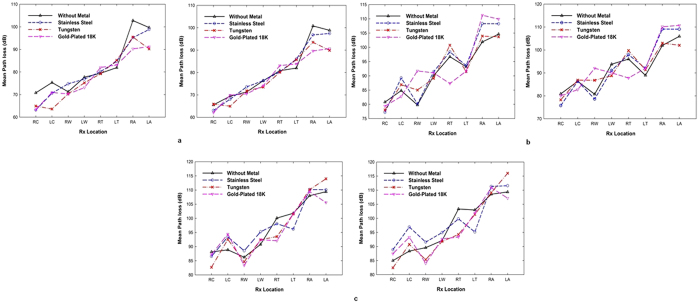
Mean path loss of Rx locations for on-body radio channel with body motion (left) and arm motion (right) when the subjects were wearing a metallic watch: (**a**) underweight; (**b**) normal; (**c**) overweight subjects.

**Figure 11:**
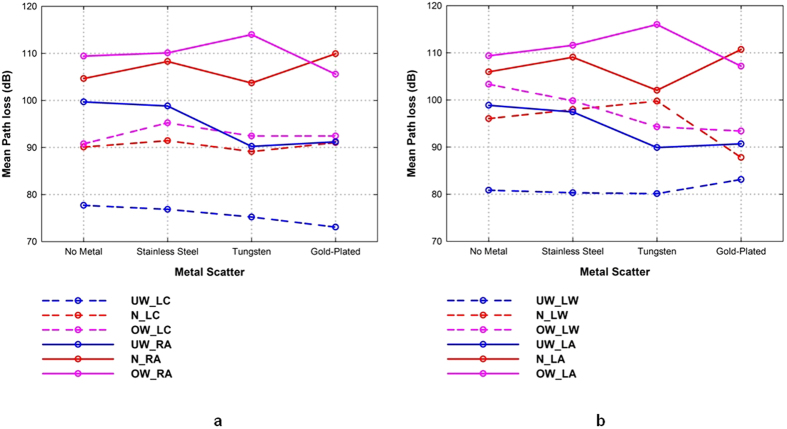
Mean path loss of dynamic on-body radio channel in the presence of metallic watches: (**a**) body motion for left chest and right ankle positions; (**b**) arm motion for left waist and left ankle positions.

To simplify the analysis, the measured path loss due to the dynamic motion of the subject was averaged over the total number of sweeps, as shown in Fig. 10(a–c). The results demonstrate that the variation of the path loss occurring at certain measurement locations for all subjects decreases when the subjects wore a gold-plated watch. As expected, quasi-variations were observed when the subjects were moving their arms in various defined ways compared to the cases where the subjects moved their bodies (including their arms) in random ways. The gold-plated watch decreases the path loss more than a tungsten watch or a stainless steel watch. The exceptions to this were at the LC for underweight subjects, and the RC and LA locations for normal and overweight subjects (*μ*_*T*_ < *μ*_*G*_) in dynamic arm movements. These results are consistent with the results for the standing-still position, where the gold-plated watch causes higher variation than the tungsten watch. To further demonstrate the relationship between body size and the effects of a metallic watch on on-body links, we compared the mean path loss for the types of metallic scatterer at on-body Rx locations that were more affected, i.e., LW and LA for body movements and RT and LA for arm movements, as shown in Fig. 11. A decreased path loss indicates a higher effect of the metallic watch on the dynamic on-body links. The results show that the effect of body movements was more related to the subjects’ height and weight for the left chest, right ankle, and left ankle positions. However, for the left waist, arm movements by the underweight subjects resulted in a smaller variation in path loss than for normal and overweight subjects (see Fig. 11(a,b)). Moreover, it is consistently observed that the on-body signal propagation for several on-body links across all groups is affected by the metal type when the arms are moving, similar to the case when the subject is standing still. A lower path loss is observed for on-body channels which transmitters were placed on the subjects’ limbs and receivers positioned closer to the metallic watch. Thus, this provides more evidence that the received signal in several radio links is dependent on the presence of loop-like metallic accessories such as watches, along with the subject specificity.

## Conclusion

In this study, the on-body radio propagation channel at 2.45 GHz in the presence of common metallic items, i.e. spectacles and watches, was investigated in a detailed and systematic way. The measurements were performed using eight realistic positions of the receiving antenna. The results showed that the spectacles had no effect on the channel in terms of path loss exponent (static case) for all subjects. This is mainly due to the fact that they were normally placed further away from the direct link. In the static scenario with watches, the results showed that the path loss characteristics for underweight subjects were more affected by the watch, up to 2%, compared to normal and overweight subjects. The path loss decreased when the metallic items were located very close to the receiving antenna, i.e., less than 20 cm.

## Additional Information

**How to cite this article**: Rahim, H. A. *et al*. Subject-Specific Effect of Metallic Body Accessories on Path Loss of Dynamic on-Body Propagation Channels. *Sci. Rep.* **6**, 29818; doi: 10.1038/srep29818 (2016).

## Figures and Tables

**Table 1:** Characteristics of the six subjects.

	UW1	UW2	N1	N2	OW1	OW2
Height (cm)	145	150	167	173	160	176
Weight (kg)	35	41	60	71	65	110
BMI	16.6	18.2	21.5	23.7	25.4	35.5
Age	25	26	26	26	24	26
Chest (cm)	75	78	83	85	96	110
Waist (cm)	66	68	80	83	89	118
Upper Arm (cm)	20	22	27	30	30	39
Wrist (cm)	14	15	16	17	17	18
Thigh (cm)	45	48	53	56	61	74
Leg (cm)	31	34	37	39	37	47
Ankle (cm)	19	21	25	25	24	26

(UW - underweight, N - normal, OW - overweight).

**Table 2:** Distance (in cm) between Tx and Rx of on-body link.

Subject	Right Chest	Left Chest	Right Waist	Left Waist	Right Thigh	Left Thigh	Right Ankle	Left Ankle
UW1	18	31	23.5	37	46	55	95	96
UW2	19	32	24	38	48	59	96	100
N1	26	40	30	46	63	71	109	112
N2	26	41	34	48	64	74	113	117
OW1	24	39	33	47	59	70	103	108
OW2	32	43	41	56	67	78	116	121

**Table 3:** Body movements done for each channel.

Measurement sweep	Movement
1	Bending left-right
2	Leaning forward
3	Rotation of torso
4	Running
5	Walking
6	Lifting things from floor
7	Exercise
8	Eating
9	Waving bye-bye
10	Moving hands near head

**Table 4:** Characterization of mean path loss for different types of metallic accessories utilizing PIFA.

Type of Metallic Accessory	Underweight			Normal			Overweight		
	*γ*	*σ* (dB)	*P*(*d*_*0*_)|dB	*γ*	*σ* (dB)	*P*(*d*_*0*_)|dB	*γ*	*σ* (dB)	*P*(*d*_*0*_)*|* dB
No spectacles/watches	4.3	0.56	53.0	4.6	0.41	58.7	4.8	0.53	59.9
Full-rimmed spectacle	4.3	0.55	53.0	4.6	0.43	58.7	4.8	0.50	59.8
Semi-rimmed spectacle	4.3	0.56	53.0	4.6	0.41	58.6	4.8	0.47	59.9
Stainless steel watch	4.3	0.56	52.9	4.6	0.42	58.7	4.8	0.48	59.9
Tungsten watch	4.3	0.59	52.7	4.6	0.41	58.7	4.8	0.48	59.9
Gold-plated 18K watch	4.3	1.34	52.1	4.6	0.40	58.7	4.8	0.47	59.9

**Table 5:** Comparison with state-of-the-art in literature.

	Underweight		Normal						Overweight				
Ref.	[34]	Present Study	[12]	[13]	[25]	[26]	[36]	Present Study	[34]	[12]	[36]	[44]	Present Study
Antenna Structure	Single-cell point source	PIFA	Tapered slot	Planar monopole	Planar monopole	Meander line	Patch	PIFA	Single-cell point source	Tapered slot	Patch	Monopole	PIFA
Polarization with respect to body surface	Tangential	Vertical	Vertical	Vertical	Horizontal	Vertical	*NA*	Vertical	Tangential	Vertical	*NA*	Normal	Vertical
Tx	LW	RUA	LW	Front of body	RUA	Arm, leg and upper part of body	LW	RUA	LW	LW	LW	Chest	RUA
Propagation scenario	LOS	NLOS	LOS	NLOS	NLOS	NLOS	NLOS	NLOS	LOS	LOS	LOS	NLOS	NLOS
Environment	Simulated	AC	AC	Large empty room	Indoor	AC	Simulated	AC	Simulated	Simulated	Simulated	AC	AC
Frequency (GHz)	2.4	2.45	3–10	3–6	3.1–10.6	3–6	2.4	2.45	2.4	3–10	2.4	2.45	2.45
*γ*	2.4	4.3	3.5	7.2	3.7	4.1	3.5	4.6	2.6	3.6	3.4	≈4.0–4.5	4.8

**Table 6:** Dimensions of the watches and conductivity of their material.

Material	Diameter (mm)	Length (mm)	Thickness (mm)	Width (mm)	Conductivity (S/m)
Stainless steel	35.9	171.0	3.9	19.2	1.10 × 10^6^
Tungsten	36.0	155.8	2.9	18.0	1.83 × 10^7^
Gold-plated 18 K	35.3	160.4	2.5	17.6	4.10 × 10^7^
